# A Verification Report of Three Meta‐Analyses in Transcranial Direct‐Current Stimulation—Motor Learning Research

**DOI:** 10.1111/ejn.70533

**Published:** 2026-05-06

**Authors:** Taym Alsalti, Ian Hussey, Malte Elson, Robert Krause, Steffi Pohl

**Affiliations:** ^1^ Leipzig University Leipzig Germany; ^2^ University of Bern Bern Switzerland; ^3^ University of Kentucky Lexington Kentucky USA; ^4^ Freie Universität Berlin Berlin Germany

**Keywords:** meta‐analysis, reproducibility, transcranial direct‐current stimulation

## Abstract

With transcranial direct‐current stimulation's (tDCS) popularity both in motor learning research and as a commercial product, it is important that the quality of evidence on its effectiveness be evaluated. Special attention should be paid to meta‐analyses, as they usually have a large impact on research and clinical practice. The aim of this verification report was to gain insight on the methodological quality of meta‐analyses estimating the effect of tDCS on motor learning. To that end, we verified the methodology of three meta‐analyses with respect to reproducibility as the main focus, and reporting quality and publication bias control as secondary aspects. The three meta‐analyses we verified largely adhered to PRISMA reporting guidelines and reported the primary effect sizes and sampling variances/confidence intervals they calculated, enabling successful reproductions of pooled effect size estimates. However, akin to previous meta‐research with similar aims, we found the methods and results sections of the meta‐analyses to be severely underreported, which compromises the ability to judge the soundness of the methodological procedure adopted as well as its reproducibility. While publication bias detection methods were applied, the approaches chosen do not allow for well informed decisions about the presence or extent of publication bias. These results reemphasise the need to transparently report methods in meta‐analyses and to meticulously evaluate their quality before and after publication.

## Graphical abstract

Out of the 47 primary effect sizes across the three meta‐analyses verified, 16 could be successfully reproduced or approximated. Thirteen effect sizes, not depicted here, could not be reproduced due to missing information.

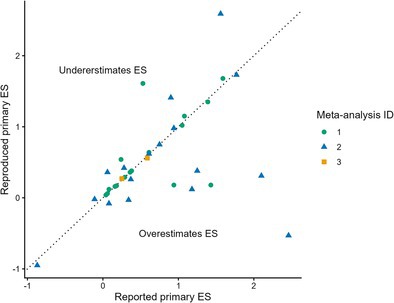

**Abbreviations**
ES: effect size
MA: meta‐analysis
PRISMA: Preferred Reporting Items for Systematic reviews and Meta‐Analyses
SD: standard deviation
SE: standard error
tDCS: transcranial direct‐current stimulation

## 1 Introduction

### 1.1 tDCS and Its Applications

Transcranial direct‐current stimulation (tDCS) is a non‐invasive brain stimulation technique which involves delivering constant, low current to the brain via electrodes fixed on the scalp (Gazzaniga et al. 2018). Although tDCS is widely believed to be safe and to cause negligible side effects, if any (Gianni et al. 2021; Nitsche et al. 2008), some studies (e.g., Bertrand et al. 2024) have conveyed reports of side effects that can compromise blinding such as itching and other bodily sensations. The typical set‐up of a tDCS protocol involves no more than a handful of inexpensive components and relatively simple steps (Gebodh et al. 2019; Woods et al. 2016). The participants receive either tDCS or get the device placed on their heads without administering any current (sham/control condition, although due to the side effects mentioned above, the blinding is often compromised) during this training.

When considering tDCS's logistic advantages, tDCS's popularity is not surprising (Buch et al. 2017). In clinical research, its efficacy for treating or attenuating depression (Brunoni et al. 2016), memory deficits in Alzheimer's patients (Bennabi et al. 2014), pain (Luedtke et al. 2012), schizophrenia (Liu et al. 2021), among other conditions, has been investigated. Interest in tDCS is not restricted to clinical settings: studies on healthy subjects have been conducted to test its effects on cognitive abilities such as language and memory (Horvath et al. 2015), affective states (Austin et al. 2016), and motor skills, such as surgery (Hung et al. 2021) and musical performance (Rosen et al. 2016).

tDCS has become so established as a research tool that it is already approved for clinical use (with some restrictions) in several countries around the world (Fregni et al. 2015). Furthermore, an ever‐richer variety of commercial tDCS products has become available on the market (Davis 2016; Wexler 2018; Zettler 2017), prompting experts (e.g., Wurzman et al. 2016) to voice concerns over the increasing prevalence of this ‘do‐it‐yourself’ use of tDCS. Out of the 449 such at‐home tDCS consumers surveyed by Wexler (2018), 52% (237) did so for enhancement purposes. tDCS devices have also become available as commercial products in many countries, often backed by weak scientific evidence to support its use. The touted benefits range from alleviating depressive symptoms to improving physical strength and dexterity. At the same time, heterogeneity in technical implementations (e.g., stimulation intensity and duration) and problems like publication bias and irreproducibility plague the tDCS literature (Buch et al. 2017). These issues likely impact meta‐analyses on this topic, which often form the basis of clinical decisions.

### 1.2 Methodological Quality of Meta‐Analyses

The popularity of tDCS might be partially driven by meta‐analyses showing positive effects of its use. Meta‐analyses are often characterised as being at the ‘top’ of the ‘evidence hierarchy’ (Evans 2003): they are cited more frequently than primary studies about the same topics, are commonly assumed to provide the most accurate estimate of an effect and have a large impact on theory development as well as policy and clinical practice (Gopalakrishnan and Ganeshkumar 2013; Gøtzsche et al. 2007; Ioannidis 2016; Lakens et al. 2017; Morganti 2007). Several papers drafting guidelines on the use of tDCS in research and the clinic extensively cite meta‐analyses (Charvet et al. 2020; Fregni et al. 2015; Lefaucheur 2016). Hence, meta‐analyses on the effects of tDCS are likely to influence its uptake in many areas. Given this, it is important to evaluate the methodological quality of these meta‐analyses as they can inform clinical practice. There are different aspects of methodological quality that can be considered, such as comprehensiveness of the study search, quality of the study screening procedure and appropriateness of statistical methods employed. Here, we focus on reporting quality, reproducibility and publication bias control.

### 1.3 Reporting Quality of Meta‐Analyses

Meta‐analysts are faced with numerous decisions with regard to which databases to search and using which strings; primary study selection and exclusion; data extraction; statistical methods, among others (Ada et al. 2012; Guzzo et al. 1987; Valentine et al. 2010; Voracek et al. 2019). Although the extent to which these methodological variations impact the conclusions of the meta‐analysis is not a completely uncontroversial issue, there is consensus regarding the importance of transparently reporting these decisions (Aguinis et al. 2011), as it is difficult to assess the quality and trustworthiness of that which one does not have access to (Page, McKenzie, et al. 2021).

Several guidelines for conducting and reporting meta‐analyses exist, for example, the Cochrane Handbook (a comprehensive guide for conducting systematic reviews and meta‐analyses, Higgins et al. 2019) or the Preferred Reporting Items for Systematic Reviews and Meta‐Analyses (PRISMA, a checklist of methodology reported items to report when conducting systematic reviews and meta‐analyses Moher et al. 2000, 2009; Page, McKenzie, et al. 2021). Although such guidelines have been available for over two decades, meta‐scientific evaluations of adherence to PRISMA and other guidelines have shown that reporting standards of meta‐analyses are generally suboptimal and that these guidelines are rarely fully adhered to (Page and Moher 2017; Polanin et al. 2020; Schalken and Rietbergen 2017). For example, while over 80% of the +80,000 systematic reviews coded by Page and Moher (2017) provided a rationale for their review, less than 60% conducted a risk of bias analysis or disclosed funding sources. Of course, neither PRISMA nor any other reporting guideline fully cover what should be reported in a meta‐analysis, but failing the basic information included in it (e.g., search strategy) indicates underreporting.

### 1.4 Meta‐Analysis Reproducibility

Given the prevalent lack of adherence to reporting checklists, it comes as no surprise that attempts to reproduce meta‐analyses often fail; the less information a meta‐analyst provides about their procedure, the harder it is to reproduce (Aguinis et al. 2011). In fact, even full adherence to reporting guidelines far from guarantees reproducibility (Weissgerber et al. 2021). Reproducibility is essential as it enables other researchers to detect errors and evaluate the defensibility of subjective choices (e.g., primary study eligibility criteria). It also facilitates updating meta‐analyses as more relevant primary studies become available (Lakens et al. 2016). Reviews of meta‐analysis reproducibility (e.g., Ford et al. 2010; Gøtzsche et al. 2007; Lakens et al. 2017; Maassen et al. 2020) emphasised somewhat different methodological aspects (e.g., complete reporting vs. computational correctness) but mostly focused on reproducing data extraction and effect size (ES) computation (both primary and pooled). Their conclusions about the reproducibility of meta‐analyses were also similar—that reproducibility of meta‐analyses was severely limited due to under‐reporting and errors—although resulting in varyingly grave consequences. For example, for four out of the eight meta‐analyses Ford et al. (2010) reviewed, the reproduced pooled ES estimates were no longer significant. Similarly, one of the meta‐analyses Gøtzsche et al. (2007) reviewed was later retracted and two were no longer significant in their reproduced versions.

### 1.5 Publication Bias Control

Publication bias describes the tendency of publishing studies that report significant results in the hypothesised direction and omit publishing non‐significant results or results that are not in line with the hypothesis. This is a major issue for meta‐analyses. Meta‐analysts can attempt to both pre‐emptively mitigate publication bias, for example, by searching the ‘grey literature’ for unpublished studies, and through the post hoc application of statistical publication bias detection and adjustment methods. Many procedures for this latter purpose have been developed in the last three decades, displaying varying performance profiles depending on assumptions made and nature of data (see, e.g., Harrer et al. 2019 for a detailed review). Besides agreement that traditional methods like Fail‐Safe *N* and Trim‐and‐Fill are not particularly informative, there is little agreement about which specific methods should be used under which conditions (Carter et al. 2019; McShane et al. 2016; Renkewitz and Keiner 2019). This necessitates transparently discussing the assumption of each test used and to what extent these assumptions are met in the current set of primary studies. Another commonly voiced recommendation is to use several methods in tandem as sensitivity analyses (see Supporting Information S1: Note 1), and not as detection or adjustment methods per se (e.g., Vevea et al. 2019).

### 1.6 Research Goals

In sum, three principal premises motivated this work: (1) tDCS appears to be remarkably popular as a research tool in basic and clinical research as well as in the form of commercial gadgets; (2) meta‐analyses of tDCS's effect on motor learning might be substantially impacting research and clinical practice and, further downstream, tDCS's uptake as a commercial product; and (3) the credibility and informativeness of meta‐analyses depend on their methodological quality. Our aim was to gain insight about the methodological quality of meta‐analyses in the tDCS motor learning literature with respect to (1) reproducibility (primary aim) as well as (2) adherence to PRISMA guidelines and (3) evaluation of and controlling for publication bias (secondary aims).

## 2 Methods

This project was originally conceived of as part of TA's master thesis. Although our methodological approach mostly followed the plan pre‐defined in the thesis proposal (accessible on the thesis' Open Science Framework [OSF] project osf.io/xyhf5), there were important deviations from the plan, especially with respect to reproducibility testing. A document listing these deviations and reasons for them can be found on the OSF project (osf.io/8w4v6). For data processing, analysis and visualisation, we used R (R Core Team 2021) and the packages dmetar (Harrer et al. 2019), dplyr (Wickham et al. 2021), ggplot2 (Wickham 2016), MAd (Hoyt and T. 2014), Matrix (Bates and Maechler 2021), meta (Balduzzi et al. 2019), metafor (Viechtbauer 2010), purrr (Henry and Wickham 2020) and tidyr (Wickham 2021). Data were extracted from figures using WebPlotDigitizer (https://wpd.starrydata2.org/, Rohatgi 2021). A video demonstration of how we extracted data from figures is available on our OSF project. All our data and code are available on the project's GitHub repository, github.com/TaymAlsalti/tDCS_meta‐analysis.

### 2.1 Sample of Meta‐Analyses

For choosing meta‐analyses, we performed a search on Google Scholar and Web of Science applying the following eligibility criteria:

- Meta‐analysis studies which quantitatively synthesise multiple (at least 3) primary studies on the effects of tDCS on motor learning.
- No restriction on primary outcomes (e.g., how speed or accuracy were measured), designs of primary studies (e.g., randomised vs. crossover designs), participants (e.g., clinical or healthy subjects) in the primary studies or any other aspect of the meta‐analysis were imposed.
- Meta‐analysis is published in English.

#### 2.1.1 Exclusion Criteria

- Reviews of any type without a quantitative synthesis
- Primary studies
- Reviews which did not report a ‘main’ meta‐analysis, but rather multiple meta‐analyses of subgroups of studies.

We unsystematically selected three meta‐analyses (Kang et al. 2016, 2018; Hung et al. 2021) which were the first to be found that (A) met our eligibility criteria and (B) had strong implications for clinical practice (all three meta‐analyses) or had a high citation rate (only the first meta‐analaysis at the time) and, thus, a high impact on the field. Since we only verified three non‐systematically selected meta‐analyses, we do not make wide‐scale generalisations about the entire field but rather illustrate strength and weaknesses of the renowned meta‐analyses in the field.

### 2.2 Reproducibility

We based our treatment of meta‐analysis reproducibility on the definition and principles of reproducibility put forward by the American Statistical Association (Broman et al. 2017): a meta‐analysis is reproducible if its authors provided enough information to go through all the necessary procedures (search, screening, data extraction, calculation of primary ESs, calculation of the pooled ES, …) to arrive at the same numerical results. However, although we acknowledge the importance of all these steps, we, like Gøtzsche et al. (2007), Lakens et al. (2017) and Maassen et al. (2020), focused on data extraction and calculation of ES estimates.

For each meta‐analysis selected, we extracted information on both the primary and meta‐analyses study levels. On the primary study level, we extracted all information necessary to calculate an ES measure as well as its standard error (SE). This included information such as sample sizes, means and SDs of outcome measures in the compared groups. We extracted these data from both the primary studies and the meta‐analyses, whenever reported. On the meta‐analysis level, we additionally extracted the pooled ES estimate across the primary studies.

#### 2.2.1 Reproducing Primary ESs

In order to reproduce the pooled ES from the meta‐analyses, it is necessary to reproduce the ESs (along with the corresponding sampling variances or SEs) from the primary studies (primary ESs) first. For classifying the reproducibility status of the primary ESs, we constructed a scheme with two variables (see Table 1): (A) whether the primary ES could be successfully reproduced numerically (results reproducibility, Goodman et al. 2016). Here, an ES was considered successfully reproduced (or reproducible) if the reproduced ES equalled the one reported in the meta‐analysis at the second decimal (e.g., 0.3334543 = 0.33) as that is how the ESs were reported in the first two meta‐analyses. We considered an ES to be approximated (but not reproduced) if the reproduced ES values were in a ±0.05 range of the reported values in the meta‐analysis. This is because we had to extract some values (e.g., means and CIs) from figures reported in the primary studies, which is likely to lead to rounding deviations if the meta‐analysts did so, too. And (B) whether the procedure we followed in reproducing the ES strictly corresponded to the information given in the meta‐analysis or to the procedure apparently adopted for at least two other ESs included in the meta‐analysis (methods reproducibility). ‘Procedure’ here includes such analytic decisions as using *p* values or test statistics in combination with sample sizes, using the raw means and SDs, using means and SDs of changes in the outcome from baseline, and so on, to estimate a standardised mean difference for a given primary study. The second variable in the classification system was thus mainly adopted to capture cases where there was a discrepancy between how the meta‐analysts reported having computed a primary ES and how they actually computed it.

**TABLE 1:** Reproducibility classification scheme for primary effect sizes.

	Reproduced ES equalled reported ES	Reproduced ES did not equal reported ES
Strictly following information given in the meta‐analysis or a standard procedure apparently adopted for at least two other, successfully reproduced (i.e., reproducible), ESs	1. Faithfully reproducible ES	2. Faithfully irreproducible ES
Following a procedure which either does not entirely correspond to the procedure the meta‐analysts report having adopted OR does not (necessarily) produce an ES that is comparable to what would result from following the procedure apparently adopted for at least two other, reproducible, ESs	3. Brute‐force reproducible ES	4. Brute‐force irreproducible ES

The distinction between ‘reproduced’ and ‘reproducible’ here is thus crucial: ‘reproduced’ means that we calculated an ES based on data extracted from the primary study that we believed the meta‐analysts might have used, whereas ‘reproducible’ primary ESs were those which numerically equalled the ones reported in the meta‐analysis. Whereas ‘faithfully’ and ‘brute‐force’ refer to whether the meta‐analysis provided enough information on how to compute the primary ES. It follows from this scheme that primary ESs that cannot be reproduced due to lack of information cannot be tested for numerical reproducibility, and thus cannot be faithfully reproducible, although they can be brute‐force reproducible. Also note that if we classify an ES as ‘faithfully irreproducible’, this does not imply that it is necessarily also brute‐force irreproducible because in most cases, we did not collect further values to test brute‐force reproducibility if the values we chose to test faithful reproducibility very clearly corresponded to the meta‐analysts' description of their procedure.

Reproducibility testing was an iterative process which involved several rounds of data extraction. The initial round of data extraction and testing reproducibility yielded few reproducible ESs as most primary studies did not report the values necessary to directly compute an ES and/or its sampling variance (e.g., for a between groups Cohen's d this would be the group means, SDs and sample sizes). Therefore, a less strict data extraction procedure was adopted, which involved the following steps:

1. For each primary ES, we first looked for the raw means and SDs of the outcome reported as having been used by the meta‐analysts. If the primary study reported multiple sets of means and SDs which can be seen as corresponding to the outcome described in the meta‐analysis (e.g., the outcome in the meta‐analysis for a given primary study is ‘Fugl‐Myer Test’ but the primary study reports values for ‘Fugl‐Myer Test ‐ upper limbs’ and ‘Fugl‐Myer Test ‐ full’), all sets were extracted.
2. If no means and SDs for the relevant outcome were reported in the primary study, means and SDs were extracted from figures. If no figures were reported which contained means and SDs (or SEs or confidence intervals [CIs], which can be converted to SDs), p and/or t values for tests on the relevant outcome were extracted, which in combination with sample sizes can be converted to ESs.
3. Based on all extracted values, we computed each primary ES using the estimator (Cohen's d or Hedges' g) reported as having been used by the meta‐analysts. If this information was not given in the meta‐analysis, we tried both formulas and for further analysis used the one which consistently approximated the reported ESs better.
4. If none of the values extracted reproduced a given ES, we double checked the correctness of the data extracted and, in some cases, extracted more values from the primary study (à la brute‐force) and computed the ES based on those.
5. We computed the sampling variances based on the ESs and the corresponding samples sizes.

The concrete procedure for data extraction thus differed for each single primary study. A detailed description of all values we extracted and how we analysed them is provided in the data analysis notebook, also available on our OSF project page.

#### 2.2.2 Reproducing the Pooled ESs

For obtaining an estimate of the pooled ES, we conducted three analyses based on different subsets of primary ES: (a) using the primary ESs and sampling variance as reported in the meta‐analysis, (b) using the faithfully reproduced and faithfully approximated in addition to the brute‐force reproduced primary ESs and (c) using only the faithfully reproduced primary ESs. This allowed us to investigate to which extent differences between reported and reproduced pooled ESs are due to problems to reproduce primary ESs and which are due to issues with the description of the meta‐analytic procedure.

### 2.3 Adherence to PRISMA Reporting Guidelines

We evaluated adherence to PRISMA reporting guidelines (Liberati et al. 2009; Moher et al. 2009) as they are the most widely adopted reporting standard for meta‐analysis. To that end, we coded whether the meta‐analysis reported the relevant information as recommended for each of the 27 items, regardless of whether the meta‐analysis reported having adhered to any reporting guidelines. Using the PRISMA checklist, we scanned the full text of each meta‐analysis, searched for keywords pertaining to each item (e.g., ‘search’ for the item ‘Search’) and noted down the item as reported regardless of how well it was described.

### 2.4 Publication Bias

We investigated in which way the meta‐analyses in our study considered the possible impact of publication bias by (a) evaluating measures taken to identify unpublished studies and (b) assessing whether and which statistical approaches were used to investigate the presence of publication bias. We additionally coded whether the meta‐analysts:

1. Searched clinical trial registries (e.g., ClinicalTrials.org)
2. Searched thesis and dissertation repositories (e.g., ProQuest)
3. Contacted known researchers in the field to inquire about unpublished results
4. Contacted authors of included studies to ask for raw data or unpublished results

Besides coding whether any statistical methods were used at all and which, we additionally tested for publication bias in each meta‐analysis using three different publication bias methods not used by the meta‐analysts to evaluate the robustness of their conclusions (see Supporting Information S1: Note 1).

### 2.5 Additional Coding/Analyses

We checked whether the meta‐analyses provided data‐analysis code, shared their extracted data, or had been pre‐registered. As outliers can heavily impact pooled ES estimates, we coded whether and how the meta‐analyses tested for the existence of outliers among the included studies and, based on the data extracted from the forest plots in the meta‐analyses, we tested for the impact of outliers on the results ourselves using the leave‐one‐out method (Harrer et al. 2021; Tobias 1999).

## 3 Results

### 3.1 Sample of Meta‐Analyses

Table 2 gives an overview of the three meta‐analyses selected. Meta‐analyses 1 and 2 aimed to estimate the effectiveness of tDCS for improving motor function in post‐stroke patients, although Meta‐analysis 2 focused exclusively on the effects of cathodal tDCS. Meta‐analysis 3 investigated effectiveness of tDCS for improving surgical performance of surgery trainees. The first meta‐analysis synthesised the results of 13 randomised controlled trials (RCTs) and four crossover trials, the second six RCTs and nine crossover trials, the third five RCTs and one crossover trial.

**TABLE 2:** Verified meta‐analyses.

General information and PRISMA adherence					Reproducibility					Publication bias			Outlier analysis	
Meta‐analysis	*N* of studies	*N* of ESs	Average sample size	Reported PRISMA items	Faithfully reproduced ESs	Faithfully reproducible ESs	Faithfully approximated ESs	Reported pooled ES	Reproduced pooled ES	Prevention measures	Statistical methods to investigate the presence of publication bias	Conclusions drawn	Reported analyses	Our analyses
Kang et al. (2016)	17	21	26	18/27	17/21	6/21	4/21	0.59	0.54	**—**	Funnel plot, Fail‐Safe *N*, trim‐and‐fill	‘Minor’	None	Pooled ES after removing 3 outliers: 0.67
Kang et al. (2018)	15	20	24	19/27	18/20	1/20	4/20	0.62	0.45	**—**	Funnel plot, trim‐and‐fill, Egger's test, Begg and Maxumdar's rank correlation test	‘Minimal’	None	Pooled ES after removing 4 outliers: 0.73
Hung et al. (2021)	6	6	32	25/27	2/6	0/6	1/6	0.66	0.42*	Searched ClinicalTrials.gov and ProQuest, contacted authors of primary studies, no restriction to English	Funnel plot, Egger's test	No conclusions drawn	Leave‐one‐out analysis. Conclusion: main results robust	Pooled ES after removing 1 outlier: 0.81

*Note*: The reproduced pooled ESs are based on meta‐analytic Model A, that is, faithfully reproduced ESs only.

* The reproduced pooled ES for MA 3 was not significantly larger than 0 since it was based on two primary studies only. Four further ESs across the three meta‐analyses count as approximated when applying a ±0.1 range instead of ±0.05.

All three meta‐analyses reported ESs in the form of standardised mean differences, although it was not consistent which group means were used (e.g., mean difference between pre and post vs. mean difference in the post values across groups). For some primary studies, Meta‐analyses 1 and 2 included more than one ES in the meta‐analysis. In Meta‐analysis 1, these represented cathodal versus sham and anodal versus sham pairs, whereas in Meta‐analysis 2, the different ESs within one study were based on two different outcomes. None of the three meta‐analyses had been pre‐registered, shared data or provided data analysis code. Meta‐analysis 3 stated that data ‘was available upon reasonable request’ (p. 11), but the corresponding author of the meta‐analysis did not respond to our email requesting more information/data. The corresponding author of the two other meta‐analyses indicated that all data are already contained in the meta‐analysis reports.

### 3.2 Reproducibility

#### 3.2.1 Reproducibility of Primary ESs

We only report on the reproducibility of the primary ESs. Note that, in general, the sampling variances were numerically reproducible whenever their corresponding primary ES were reproducible. Most primary ESs in Meta‐analyses 1 and 2 (17/21 or ~81% and 18/20 or ~85%, respectively) and 2/6 or 33% in MA 3 could be faithfully reproduced (see Table 2). That is, (a) enough information was available or inferable from the meta‐analyses and (b) seemingly appropriate data were reported in the corresponding primary studies, to attempt to recalculate about 77% of all primary effect sizes across the three meta‐analyses. Of those, 10 (~48%), 5 (25%) and 1 (16%), respectively, were reproducible (i.e., successfully reproduced numerically) or approximated.

We faced considerable difficulties in reproducing the primary ESs for all three meta‐analyses, mainly due to limited reporting of their methods sections. The process necessitated several rounds of data extraction and testing. It was in most cases impossible to know from the paper how the meta‐analysts calculated each primary ES and in a brute‐force procedure, we had to rely on trial and error to figure out which values from the primary studies were used. Table 3 lists the relevant pieces of information which were reported or missing from the three meta‐analyses. As can be seen from Table 3, while sample sizes and groups being compared were reported by all three meta‐analyses, none of them reported enough information on the outcome and exact calculation method to ensure unambiguity.

**TABLE 3:** Reproducibility related elements and whether each meta‐analysis (MA) provided them.

Reproducibility related element	MA 1	MA 2	MA 3
Extracted data	—	—	—
Data analysis code	—	—	—
Primary ESs (e.g., in a tree plot)	+	+	+
Sampling variances of the ESs	—	—	—
CIs for the ESs	+	+	+
Pooled ES	+	+	+
Type of calculated ES (e.g., Hedges' *g*)	—	—	+
Sample sizes	+	+	+
Outcome measure for each ES (e.g., ‘Total latency score in JHFT’)	+	+	—
Enough details about the outcome used so as to leave no room for ambivalence (e.g., ‘Upper Limb FMA’ instead of just ‘FMA’ when the primary study reported both ‘Upper Limb FMA’ and ‘Total’)	—	—	—
Rationale for choosing that specific outcome measure	—	—	+
What the two compared groups were (e.g., ‘CG: sham before intervention, TG: ctDCS on cH after intervention’)	+	+	+
Primary study design (e.g., RCT, crossover)	—	—	—
ES and sampling variance/SE formulas for the different designs	+	—	—
What the reported sample size denotes depending on study design (e.g., group vs. total *N*)	—	—	—
Which type of values were used to compute each ES (e.g., means and SDs vs. *p* value and *n*s)	—	—	—
Which exact values were used and where they were found (e.g., *p* value reported on page *x* line *y* or means and SDs reported in Figure *z*)	—	—	—
Software used to run the meta‐analysis	+	+	+
Fixed versus random effects model	+	+	+
Which between‐study heterogeneity estimator was used	—	—	—

*Note*: + indicates that the respective information was reported for all primary studies that it was not reported for all primary studies.

**FIGURE 1:**
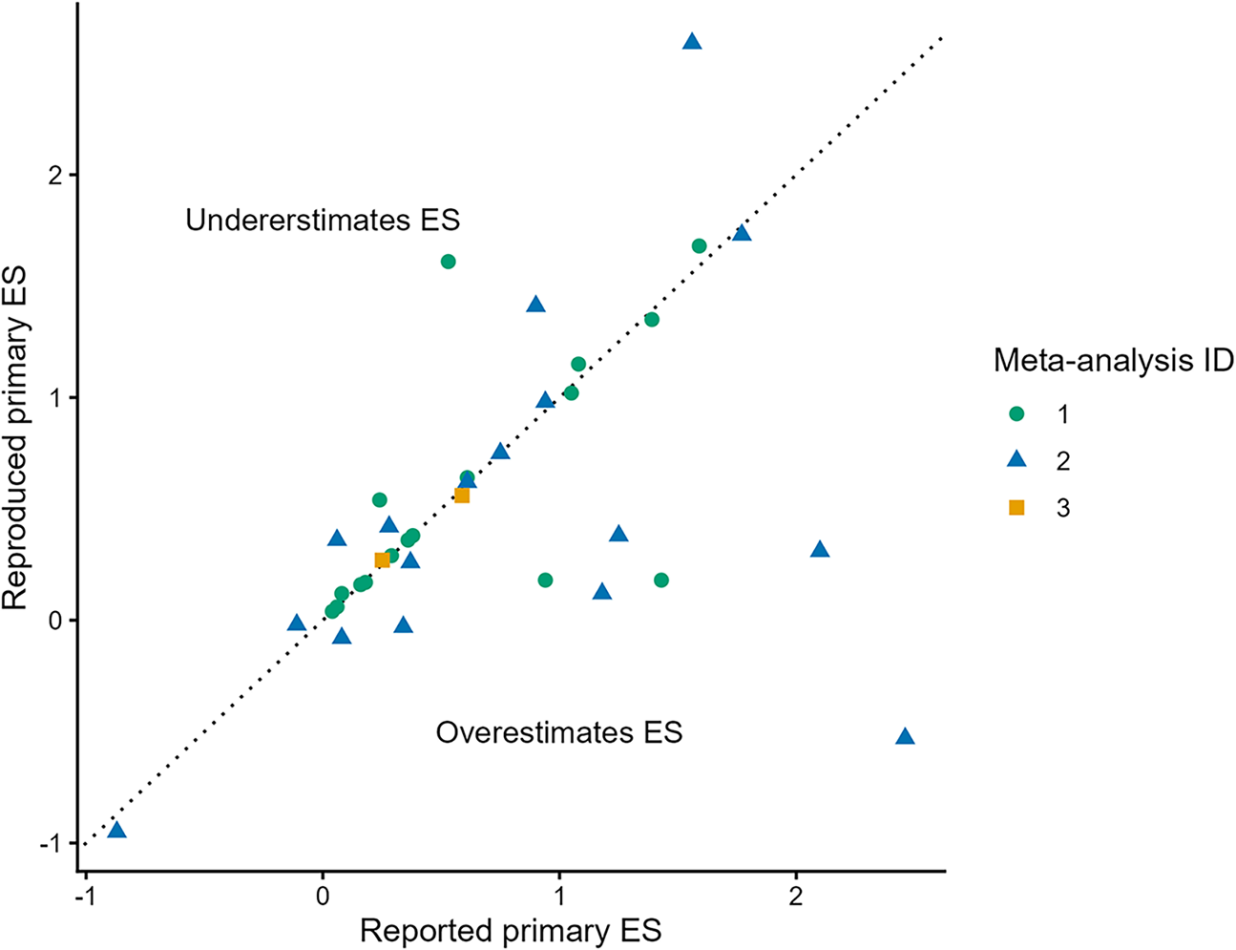
Comparison of ESs reported in the three meta‐analyses and their faithfully reproduced counterparts. The 13 ESs that could not be faithfully reproduced are not depicted (see Figure S1 for an equivalent plot showing both faithfully and brute‐force reproduced ESs).

In most cases, it was unclear why we failed to reproduce any given primary ES, but our extensive attempts at brute‐force reproducing certain primary ESs which ended up exactly reproducing reported ESs gave us some insights for reasons why we could not faithfully reproduce these ESs: For example, all three meta‐analyses used values belonging to different outcomes than specified and used *p* values from nonparametric tests (e.g., median tests). The third MA used *p* values expressed as a range (e.g., *p* < 0.01). Tables S1–S3 list all reproduced‐reported primary ES pairs for all three meta‐analyses, their corresponding reproducibility classification, and the potential reason for irreproducibility, if applicable. Figure 1 depicts all primary ESs reported in the forest plots shown in three meta‐analyses and how they compared to their reproduced counterparts. Across all three meta‐analyses, reproduced ESs were on average 0.18 smaller than reported ones (max = 1.08, min = −2.99). The average absolute difference was 0.37 (max = 2.99, min = 0).

#### 3.2.2 Reproducibility of Pooled ESs

Since all three meta‐analyses reported having fit a random‐effects model using the Comprehensive Meta‐Analysis software, which per default estimates between‐study heterogeneity via the Der‐Simonian‐Laird method (DerSimonian and Laird 1986), we used these settings for all our analyses, too. Whereas the pooled ESs we calculated using the primary ESs reported in the meta‐analyses and the sampling variances extracted from funnel plots or converted from CIs were reproducible, those based on faithfully reproduced primary ESs were on average 0.15 points smaller than the reported ones. The largest impact was suffered by the already small MA3, whose pooled ES was not significant based on the two faithfully reproduced ESs. When adding the brute‐force reproduced ESs to the faithfully reproduced ones, we got pooled ESs of 0.70, 0.40 and 0.65, respectively, for the three meta‐analyses.

### 3.3 PRISMA Adherence

All three meta‐analyses reported most of the items given in the PRISMA guidelines. Meta‐analyses 1 and 2 did not report having adhered to any reporting guidelines. Despite this, they can be seen as having reported the content of 18 and 19 items, respectively, out of the 27 PRISMA (Moher et al. 2009) items. Meta‐analysis 3 reported having adhered to the most recent PRISMA guidelines (Page, McKenzie, et al. 2021) but we evaluated the adherence to the items of the 2009 version to ensure comparability to the other meta‐analyses. Meta‐analysis 3 reported the content of 25 out of the 27 items. To highlight one aspect, all three meta‐analyses adequately described their eligibility criteria but none of them described the actual process of how the criteria were enforced.

### 3.4 Publication Bias

Table 2 gives an overview of measures taken by the meta‐analysts to investigate or control for publication bias. All three meta‐analyses mentioned publication bias and applied a number of different tests to investigate the presence of publication bias. They either concluded that there is hardly any bias or did not draw a conclusion at all. The authors of meta‐analysis 1 concluded that the findings of the tests they used ‘support a minor publication bias conclusion’ (Kang et al. 2016, 348). Similarly, the conclusion in meta‐analysis 2 (Kang et al. 2018, 5) was ‘minimal publication bias in the studies used’. No clear conclusion was provided in Meta‐analysis 3. Of all three meta‐analyses, only Meta‐analysis 3 reported having taken measures to pre‐emptively mitigate its effects. These included a number of different measures aiming at identifying unpublished studies.

### 3.5 Additional Coding/Analyses

Only the third meta‐analysis mentioned outliers or influential studies. They ran a leave‐one‐out analysis and reported that the main results did not change due to removing any one of the six studies they included. Our leave‐one‐out analysis indicated the presence of influential studies in all three meta‐analyses (see Figures S2–S4).

Attempting to reproduce the meta‐analyses revealed further methodological issues which do not directly pertain to the main methodological aspects we investigated: All three meta‐analyses indiscriminately combined primary studies of different designs. For example, they computed ESs using the same formula for both controlled and crossover designs, a procedure which neglects bias resulting from estimating sampling variances for crossover studies without accounting for carry‐over effects or correlations between time points (Borenstein and Hedges 2019; Madeyski and Kitchenham 2018; Morris and DeShon 2002). Furthermore, the fact that the meta‐analysts used the total sample size of the cross‐over trial to replace the treatment and control group sample sizes is likely to have inflated the power of these pair‐wise comparisons (which is especially relevant for the publication bias tests). Another issue the meta‐analysts neglected to account for is ES dependency (Gleser and Olkin 2009), which is especially critical in the case of the first two meta‐analyses as they derived multiple ESs from single studies.

## 4 Discussion

The aim of this work was to evaluate the methodological quality of three impactful meta‐analyses that we selected from the field of tDCS‐motor learning research. We found that although the meta‐analyses largely fulfilled reporting requirements like PRISMA (Moher et al. 2009), they were too underreported to allow for smooth reproductions. While pooled ESs were reproducible based on values extracted from tables or figures (and default software settings for heterogeneity estimators), a considerable number of primary ESs were not. The reported primary ESs were on average larger than the ones reproduced. Consequently, we failed to numerically reproduce the pooled ES estimates reported in all meta‐analyses when following the procedures they described. As to publication bias control, only Meta‐analysis 3 reported having searched the grey literature. While all three meta‐analyses used several publication‐bias detection methods, they mostly used ‘traditional’ approaches without discussion of their assumptions or appropriateness.

The most notable finding is probably the high prevalence of discrepancies between how the meta‐analysts reported having computed individual ESs and how they apparently did it. These discrepancies were most often in relation to the outcomes used. For example, there were multiple cases where the meta‐analysis reported having used an outcome *X*, whereas they seemed to have used the outcome change in *X* from baseline. This was particularly perplexing when values for both outcome and outcome change were reported in the primary study. In general, all primary studies included in the meta‐analyses reported values/tests for several outcomes and Meta‐analysis 3 was the only one to provide a rationale, albeit a vague one (i.e., that they used the outcome the primary study defined as their primary outcome), for why they chose the outcome they did for each primary study. In most cases, it was impossible to infer how these things came about as the authors of Meta‐analyses 1 and 2 did not respond to our request for data or data analysis code or protocol and the authors of Meta‐analysis 3 did not respond to our email at all. On the whole, we had similar difficulties in our reproducibility endeavour as Lakens et al. (2017) and Maassen et al. (2020).

Although our reproduced pooled ESs were calculated using less primary ESs for all three meta‐analyses (due to methodologically irreproducible ESs), none of our reproductions led to a radical change in the pooled ES estimate like in Gøtzsche et al.'s (2007) or Ford et al.'s (2010) reviews. For example, none of the pooled ES estimates was completely nullified or changed its sign in the reproduced version. However, we documented several haphazard ways some primary ESs were calculated as well as some errors. Given this, a reproduction that is compliant with methodological guidelines rather than attempting to be faithful to the meta‐analysts' workflow might very well have led to different results. There are some indications that the meta‐analysts' deviations from their procedure led to overestimating the pooled ESs.

### 4.1 Recommendations

Although the meta‐analyses we reviewed verified were adequate in some respects (e.g., they all reported a forest plot displaying all ESs and their CIs), there is clearly some room for improvement. As a remedy for the compromised reporting quality and reproducibility, more detailed descriptions of the methodological procedure are called for, ideally accompanied by raw data and the data analysis code (Lakens et al. 2016; Page, Moher, et al. 2021). As publication bias may heavily impact the results of meta‐analyses, we recommend to (a) take measures to control for publication bias (Vevea et al. 2019) and (b) apply a more extensive testing procedure involving sensitivity analyses using different methods and different parameter settings within a method and discussion of the respective assumptions. When dealing with a highly heterogeneous set of ESs, van Aert et al. (2016) recommend splitting the set into subgroups based on theoretical and methodological considerations before carrying out the bias testing procedure. Inzlicht et al. (2015) recommend presenting a range of plausible pooled ESs based on different publication bias tests (using different assumptions).

No self‐evident solutions can be offered for the shortcomings related to the synthesis‐related aspects of the meta‐analyses. There is immense variation in reporting quality of primary studies and meta‐analysts usually have no other choice but to work with the data reported in the primary study (i.e., when contacting the authors of the primary study for more data is not feasible or proves to be fruitless, Higgins et al. 2019). It is thus entirely understandable that meta‐analysts must sometimes resort to alternative means of calculating certain measures. However, there are better ways to do this, too, than what the meta‐analysts seemingly opted to do. For example, instead of converting a p‐value derived from a medians test to an ES, the meta‐analysts could have estimated the means and SDs based on the medians and the corresponding interquartile ranges and calculated the ES based on these means and SDs, which would have yielded a more comparable result to an ES calculated based on actual means and SDs (Hozo et al. 2005; Wan et al. 2014). Similarly, there are methods to compute the ESs and their corresponding sampling variances when dealing with crossover studies (Madeyski and Kitchenham 2018). Estimating the ESs using the formula for within‐subjects designs would have taken the correlations between time points into account (Borenstein and Hedges 2019).

### 4.2 Limitations

There are several limitations of our study. First, we defined our exclusion criteria based on largely practical considerations. Their high restrictiveness has probably led to a sample of meta‐analyses that is not representative of the field at large. Our non‐systematic literature search and study selection strategy can only have exacerbated this issue. It is also possible that our eligibility criteria correlated with the quality of the meta‐analyses we reviewed. Clearly, given that we only verified three non‐systematically selected meta‐analyses, no generalisations to the field at large are possible. Nevertheless, the results give us information on the reproducibility of some meta‐analyses in the field. Second, although we had a mechanism in place to minimise the probability of data extraction errors on our part when evaluating reproducibility, it cannot be excluded that potential errors when extracting data for other variables (e.g., for PRISMA adherence) influenced our results as data extraction and coding was not checked by others (Buscemi et al. 2006; Jones et al. 2005).

Third, we focused on reproducibility of data extraction and calculation. Our study did not investigate reproducibility of the search for primary studies. This, is however, also an important aspect of reproducibility. Fourth, throughout the process of ES reproduction, we had to make subjective decisions that cannot be guaranteed to have been faultless. Notably, it was not always trivial to judge whether our procedure for reproducing a certain primary ES strictly followed the procedure purported to have been used by the meta‐analysts. For example, we managed to approximate ES No. 5 in Meta‐analysis 3 by averaging the means and SDs of two outcomes and computing an ES based on the average value. We subsequently classified this ES as faithfully reproducible because the meta‐analysts reported having used what each primary study defined as its primary outcome and this specific primary study defined both these outcomes as its primary outcomes. However, since the meta‐analysts did not provide any information on how they computed the ES or any indication that they took an average, it is almost certain that they calculated the ES differently because otherwise we would have successfully reproduced the ES to the third decimal like we did the other brute‐force reproducible ones. Other such examples are documented in the reproducibility report available on the project's GitHub repo (taymalsalti.github.io/tDCS_meta‐analysis/02_reproducibility_report.html).

Future similar works may hence aim for a more fine‐grained and nuanced evaluation of reporting quality which goes beyond the minimal requirements set by reporting guidelines, a more comprehensive reproducibility testing which is not restricted to data extraction and ES calculation as well as a thorough investigation of robustness towards changes in analytical decisions (especially data selection and outcomes used), and a more principled approach towards evaluating publication bias assessment.

### 4.3 Implications

All in all, our results indicate that the methodological limitations prevalent in meta‐analyses in neighbouring fields can also be observed in some meta‐analyses studying the effect of tDCS on motor learning. Given the high status that meta‐analyses enjoy on the ‘hierarchy of evidence’ (Evans 2003), they are likely to be influential both within and beyond the restricted realm of scientific publishing. For example, clinicians could rely on them in informing their decisions about treating patients.

The three meta‐analyses we verified have been cited over 350 times (333, 31 and 18 times, respectively, as of 28.11.2025, according to Google Scholar), which indicates they might already be heavily influencing researchers and other parties interested in tDCS effects. The first meta‐analysis was cited by Lefaucheur et al. (2017), one of the papers outlining guidelines for the clinical use of tDCS we cited above. We argue that the results of such meta‐analyses need to be viewed with caution and in light of their limitations. Clinical guidelines citing evidence reported by such meta‐analyses should appropriately caveat such citations for practitioners with detailed and accessible discussions about the uncertainties arising from their limitations. Transparent reporting and discussion of limitations on the part of meta‐analysts would facilitate the task of evaluating the strength of the evidence they deliver.

### 4.4 Conclusions

We hope to have provided consumers of tDCS‐motor learning research with an incentive to evaluate meta‐analyses in this field more critically when making decisions about the use of tDCS and meta‐analysts with aspects to consider when conducting meta‐analyses in the future. By no means do we want to imply that the solutions presented above are easy. Despite the abundance and comprehensiveness of guidelines and tutorials on how to conduct a transparent and reproducible meta‐analysis (e.g., Moreau and Gamble 2022; Quintana 2015), it is undeniable that meticulous adherence to guidelines and making one's meta‐analysis reproducible require a substantial amount of time and effort. Likewise, it cannot be expected from substantive researchers to be adept at every methodological/statistical aspect related to conducting a meta‐analysis.

Guidelines which also include advancements in statistical procedures for controlling publication bias, may aid researchers in conducting meta‐analyses (e.g., Vevea et al. 2019). Placing more emphasis on flagship open science practices such as pre‐registration and data and code sharing (Maassen et al. 2020; Page, Moher, et al. 2021), may also help advance meta‐analysis. Providing data and code facilitates reanalysis of the data and allows for analyses that investigate the impact of different methodological decisions and sensitivity of the results to them (Taylor and Munafò 2016; e.g., Voracek et al. 2019). Journals can be advised to require pre‐registrations of meta‐analyses or employing specialised statistical reviewers (Hardwicke et al. 2019).

Finally, since the robustness of the results of a meta‐analysis is related to the quality of primary studies it synthesises, meta‐analysts cannot be expected to carry all the responsibility for improvement (Aguinis et al. 2011; Borenstein et al. 2009). In the quest for a reliable cumulative science, efforts to counteract or alleviate the methodological issues found in the tDCS primary literature (e.g., neglected heterogeneity in employed tDCS parameters, compromised reproducibility due to incomplete reporting; Buch et al. 2017) must also be supported. Such efforts include checklists outlining all aspects which should be disclosed when reporting the results of a tDCS study (Buch et al. 2017) and statistical models which can be used to systematically determine the optimal tDCS parameters to use depending on the specific setting (Lipka et al. 2021). A more coherent primary body of literature will likely result in more informative synthesis.

## Author Contributions

**Taym Alsalti:** conceptualization, data curation, formal analysis, investigation, methodology, project administration, software, visualization, writing – original draft, writing – review and editing. **Ian Hussey:** conceptualization, visualization, writing – review and editing. **Malte Elson:** conceptualization, writing – review and editing. **Robert Krause:** conceptualization, formal analysis, writing – review and editing. **Steffi Pohl:** conceptualization, formal analysis, validation, writing – review and editing.

## Funding

This project was not funded.

## Ethics Statement

The authors have nothing to report.

## Consent

The authors have nothing to report.

## Conflicts of Interest

The authors declare no conflicts of interest.

## Supporting information


**Table S1:** *Reproducibility of primary SMDs, Meta‐analysis 1*.
**Table S2:** Reproducibility of primary SMDs, Meta‐analysis 2.
**Table S3:** Reproducibility of primary SMDs, Meta‐analysis 3.
**Figure S1:** including brute‐force reproduced SMDs.
**Figure S2:** Outlier analysis, meta‐analysis 1. ESs 6, 7, and 8 contribute disproportionately to both the variance and the pooled ES. This of course does not necessarily mean that these outlying effect sizes are wrong, rather only that they differ considerably from the other effect sizes and likely drive the pooled effect sizes to a much larger extent than the others. Their inclusion in the final meta‐analysis must be more transparently justified.
**Figure S3:** Outlier analysis, meta‐analysis 2. ES 19 contributes disproportionately to both the variance and the pooled ES. ESs 8 and 9 mostly to the pooled ES.
**Figure S4:** Outlier analysis, meta‐analysis 3. ES 2 contributes disproportionately to both the variance and the pooled ES.

## Data Availability

Available under https://github.com/TaymAlsalti/tDCS_meta‐analysis/tree/main/data.
