# Supplementary material for: A Verification Report of Three Meta‐Analyses in Transcranial Direct‐Current Stimulation—Motor Learning Research
Source: Eur J Neurosci. 2026 May 6;63:e70533. doi: 10.1111/ejn.70533 (PMC13147320; doi:10.1111/ejn.70533)
Supplement: Supplementary file 1 — Table S1: Reproducibility of primary SMDs, Meta‐analysis 1. Table S2: Reproducibility of primary SMDs, Meta‐analysis 2. Table S3: Reproducibility of primary SMDs, Meta‐analysis 3. Figure S1: including brute‐force reproduced SMDs. Figure S2: Outlier analysis, meta‐analysis 1. ESs 6, 7, and 8 contribute disproportionately to both the variance and the pooled ES. This of course does not necessarily mean that these outlying effect sizes are wrong, rather only that they differ considerably from the other effect sizes and likely drive the pooled effect sizes to a much larger extent than the others. Their inclusion in the final meta‐analysis must be more transparently justified. Figure S3: Outlier analysis, meta‐analysis 2. ES 19 contributes disproportionately to both the variance and the pooled ES. ESs 8 and 9 mostly to the pooled ES. Figure S4: Outlier analysis, meta‐analysis 3. ES 2 contributes disproportionately to both the variance and the pooled ES. [file EJN-63-0-s001.docx]

Supplemental Materials to the manuscript:
A Methodological Evaluation of Meta-Analyses in tDCS - Motor Learning Research

Table S1
 *Reproducibility of primary SMDs, Meta-analysis 1*

| SMD no. | Reported SMD | Reproduced SMD | Reproducibility classification | Reason for irreproducibility or approximation |
| --- | --- | --- | --- | --- |
| 1 | 0.16 | 0.16 | Faithfully reproducible | Not applicable. |
| 2 | 0.18 | 0.17 | Faithfully approximated | Values extracted from figure. Crossover design. Used total sample size for both the control and treatment group sample sizes. |
| 3 | 0.36 | 0.36 | Faithfully reproducible | Not applicable. |
| 4 | 0.08 | 0.12 | Faithfully approximated | Values extracted from figure. |
| 5 | 0.38 | 0.38 | Faithfully reproducible | Not applicable. |
| 6 | 0.04 | 0.04 | Faithfully reproducible | Not applicable. |
| 7 | 0.06 | 0.06 | Faithfully reproducible | Not applicable. |
| 8 | 1.59 | 1.68 | Faithfully irreproducible | Not inferable. |
| 9 | 1.08 | 1.15 | Faithfully irreproducible | Not inferable. |
| 10 | 1.05 | 1.02 | Faithfully approximated | Values extracted from figure. |
| 11 | 1.39 | 1.35 | Faithfully approximated | Values extracted from figure. |
| 12 | 0.93 | NA | Brute-force irreproducible | Not inferable. Crossover design. |
| 13 | 0.82 | NA | Brute-force irreproducible | Not inferable. Crossover design. |
| 14 | 0.29 | 0.29 | Faithfully reproducible | Not applicable. |
| 15 | 0.61 | 0.64 | Brute-force approximated | Approximated using percentage change from baseline values. |
| 16 | 1.43 | 0.18 | Faithfully irreproducible, Brute-force reproducible | Outcome used does not correspond to description. |
| 17 | 0.94 | 0.18 | Faithfully irreproducible | Not inferable. |
| 18 | 0.24 | 0.54 | Faithfully irreproducible | Not inferable. |
| 19 | 0.65 | NA | Brute-force reproducible | Outcome used does not correspond to description. |
| 20 | 0.72 | NA | Brute-force reproducible | Successfully reproduced using a p-value derived from a medians test. |
| 21 | 0.53 | 1.61 | Faithfully irreproducible, brute-force irreproducible | Not inferable. |

Table S2

*Reproducibility of primary SMDs, Meta-analysis 2*

| SMD no. | Reported SMD | Reproduced SMD | Reproducibility classification | Reason for irreproducibility/ approximation |
| --- | --- | --- | --- | --- |
| 1 | 0.96 | NA | Faithfully irreproducible | Not inferable. Crossover design. |
| 2 | 2.46 | -0.53 | Faithfully irreproducible | Not inferable. Crossover design. |
| 3 | 0.68 | NA | Brute-force reproducible | Outcome used does not correspond to description. Crossover design. |
| 4 | 1.56 | 2.59 | Faithfully irreproducible | Not inferable. Crossover design. |
| 5 | 1.25 | 0.38 | Faithfully irreproducible | Not inferable. Crossover design. |
| 6 | 0.28 | 0.26 | Faithfully approximated | Not applicable. Values extracted from figure. |
| 7 | 0.06 | 0.17 | Faithfully irreproducible | Not inferable. |
| 8 | -0.14 | NA | Brute-force reproducible | Successfully reproduced using a p-value derived from a Kruskal-Wallis test of differences between the three groups anodal, cathodal, and sham. |
| 9 | -0.11 | -0.02 | Faithfully irreproducible, Brute-force reproducible | Successfully reproduced using a p-value derived from a Kruskal-Wallis test of differences between the three groups anodal, cathodal, and sham. Means and SDs were reported in the primary study for the outcome used. |
| 10 | 0.94 | 0.98 | Faithfully approximated | Not applicable. Values extracted from figure. |
| 11 | 1.77 | 1.73 | Faithfully approximated | Not applicable. Values extracted from figure. |
| 12 | 0.37 | 0.26 | Faithfully irreproducible | Not inferable. |
| 13 | 0.34 | -0.03 | Faithfully irreproducible | Not inferable. Crossover design. |
| 14 | 2.10 | 0.31 | Faithfully irreproducible | Not inferable. |
| 15 | 1.18 | 0.12 | Faithfully irreproducible | Not inferable. |
| 16 | 0.08 | -0.08 | Faithfully irreproducible | Wrong sign. |
| 17 | 0.75 | 0.75 | Faithfully reproducible | Not applicable. |
| 18 | 0.61 | 0.62 | Faithfully approximated | Not applicable. Crossover design. Values extracted from figure. |
| 19 | -0.87 | -0.95 | Faithfully irreproducible | Not applicable. Crossover design. |
| 20 | 0.90 | 1.41 | Faithfully irreproducible | Not inferable. Crossover design. |

Table S3
*Reproducibility of primary SMDs, Meta-analysis 3*

| SMD no. | Reported SMD | Reproduced SMD | Reproducibility classification | Reason for irreproducibility |
| --- | --- | --- | --- | --- |
| 1 | 0.84 | NA | Brute-force reproducible | Successfully reproduced using a p-value (reported in the primary study as a range “<0.01”) derived from a difference in medians test. |
| 2 | 0.25 | 0.27 | Faithfully irreproducible, Brute-force approximated | Successfully approximated using the values for one of the two outcomes indicated to have been used and doubling the tDCS group sample size. |
| 3 | 0.98 | NA | Brute-force reproducible | Sucessfully reproduced using a p-value (reported as a range “<0.01”) derived from a medians test in combination with the total sample size in place of both treatment and control group sample sizes. Notably, this was not a journal article, but a conference abstract. |
| 4 | 1.19 | NA | Brute-force reproducible | Outcome used does not correspond to description. |
| 5 | 0.59 | 0.56 | Faithfully approximated | Approximated by averaging two sets of means and SDs for two different outcomes. Although the meta-analysts did not describe having done this, we tenuously classified this reproduction as faithful because both outcomes were defined as primary outcomes in the primary study and the meta-analysts wrote that they used whatever the primary studies defined as primary outcomes. |
| 6 | 0.58 | NA | Brute-force reproducible | Successfully reproduced using a p-value based on difference between tDCS and sham groups in change from baseline |


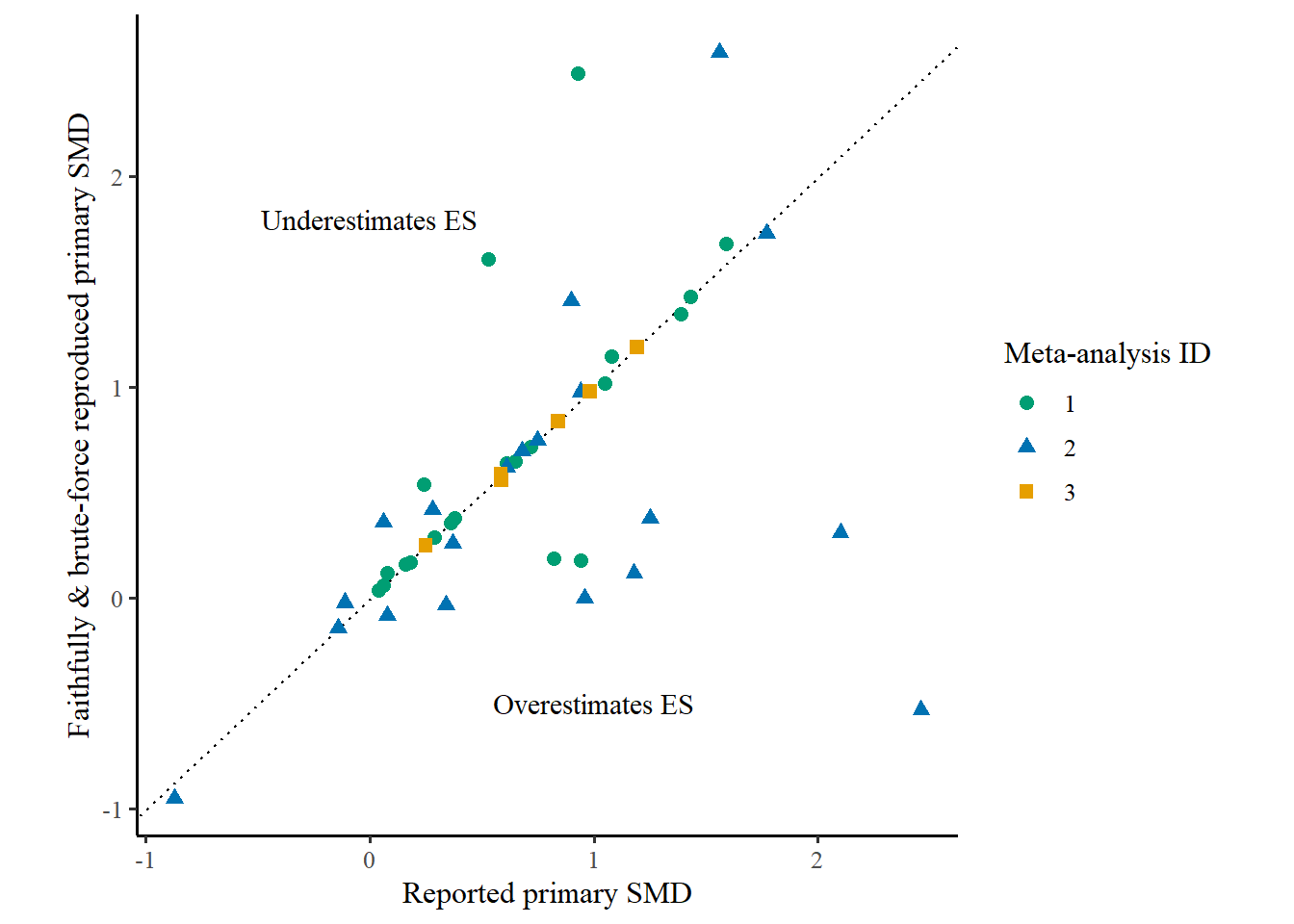


Figure S1 including brute-force reproduced SMDs

## Supplementary Note 1

Besides coding whether any statistical methods were used at all and which, we tested for publication bias in each meta-analysis using 3 different methods: PET-PEESE (T. Stanley, 2008; T. D. Stanley & Doucouliagos, 2014), $p$-curve (Simonsohn et al., 2014b, 2014a), and the three-parameter selection model (McShane et al., 2016). For these analyses, we used the ESs and the sample sizes reported in the meta-analyses along with the sampling variances extracted from the funnel plots in the case of the first two meta-analyses and calculated based on the CIs in the case of the third meta-analysis. We chose these three methods specifically for practical reasons and not because they are necessarily better than alternatives (the *p*-curve especially has received strong criticism, see Morey & Davis-Stober, 2025): they are implemented in already available R packages (see <https://taymalsalti.github.io/tDCS_meta-analysis/03_pub-bias_outlier_analyses.html> for more details).

For meta-analysis 1, the estimate of the true effect produced by the three-parameter selection model (0.59) was virtually identical to the original. The estimate produced by the $p$-curve was larger (0.70). Only the PET-PEESE intercepts (0.34 and 0.45, respectively) indicated that the random-effects model-based estimates might be overestimating the true effect. For meta-analysis 2, the selection model (0.20), $p$-curve (0.18), and PEESE (0.21) estimates were much smaller than the original (0.62). The PET intercept (-0.12) was negative. Similar results were observed for the last meta-analysis: the estimates produced by the the $p$-curve and PET-PEESE were 0.40, -0.71, and 0.01, respectively. Only the selection model yielded an estimate which is close to the one based on the random-effects model (0.62). These results indicate that the meta-analysts’ conclusion that publication bias is not a concern are not robust, but rather sensitive to the specific method used.

**References**

McShane, B. B., Böckenholt, U., & Hansen, K. T. (2016). Adjusting for Publication Bias in Meta-Analysis: An Evaluation of Selection Methods and Some Cautionary Notes. *Perspectives on Psychological Science*, *11*(5), 730–749. https://doi.org/10.1177/1745691616662243

Morey, R. D., & Davis-Stober, C. P. (2025). On the Poor Statistical Properties of the P-Curve Meta-Analytic Procedure. *Journal of the American Statistical Association*, *0*(0), 1–13. https://doi.org/10.1080/01621459.2025.2544397

Simonsohn, U., Nelson, L. D., & Simmons, J. P. (2014a). P-curve: A key to the file-drawer. *Journal of Experimental Psychology: General*, *143*(2), 534–547. https://doi.org/10.1037/a0033242

Simonsohn, U., Nelson, L. D., & Simmons, J. P. (2014b). p-Curve and Effect Size: Correcting for Publication Bias Using Only Significant Results. *Perspectives on Psychological Science: A Journal of the Association for Psychological Science*, *9*(6), 666–681. https://doi.org/10.1177/1745691614553988

Stanley, T. (2008). Meta‐Regression Methods for Detecting and Estimating Empirical Effects in the Presence of Publication Selection*. *Oxford Bulletin of Economics and Statistics*, *70*(1), 103–127.

Stanley, T. D., & Doucouliagos, H. (2014). Meta-regression approximations to reduce publication selection bias. *Research Synthesis Methods*, *5*(1), 60–78. https://doi.org/10.1002/jrsm.1095

## Outlier analyses


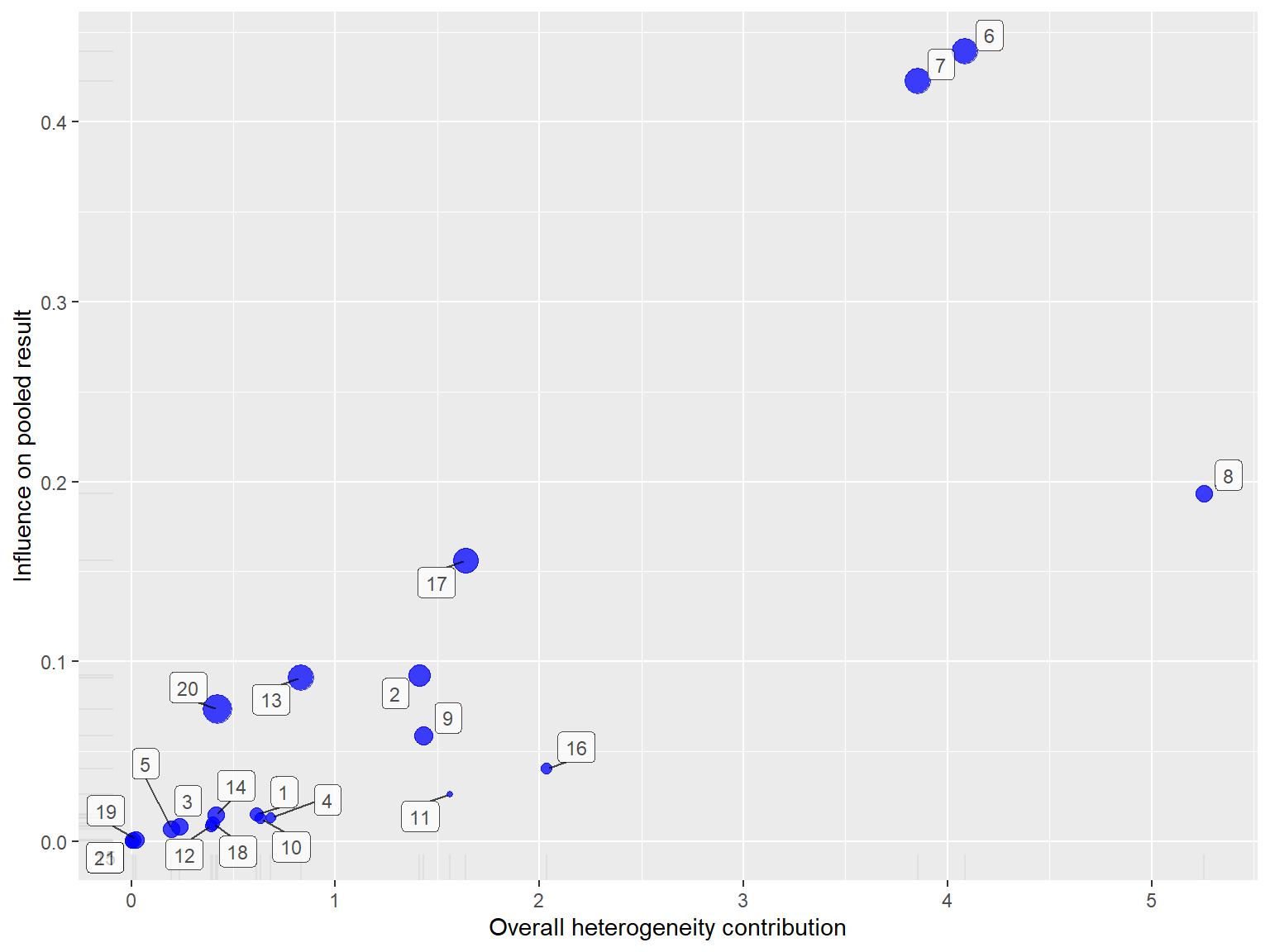


Figure S2 Outlier analysis, meta-analysis 1. ESs 6, 7, and 8 contribute disproportionately to both the variance and the pooled ES. This of course does not necessarily mean that these outlying effect sizes are wrong, rather only that they differ considerably from the other effect sizes and likely drive the pooled effect sizes to a much larger extent than the others. Their inclusion in the final meta-analysis must be more transparently justified.


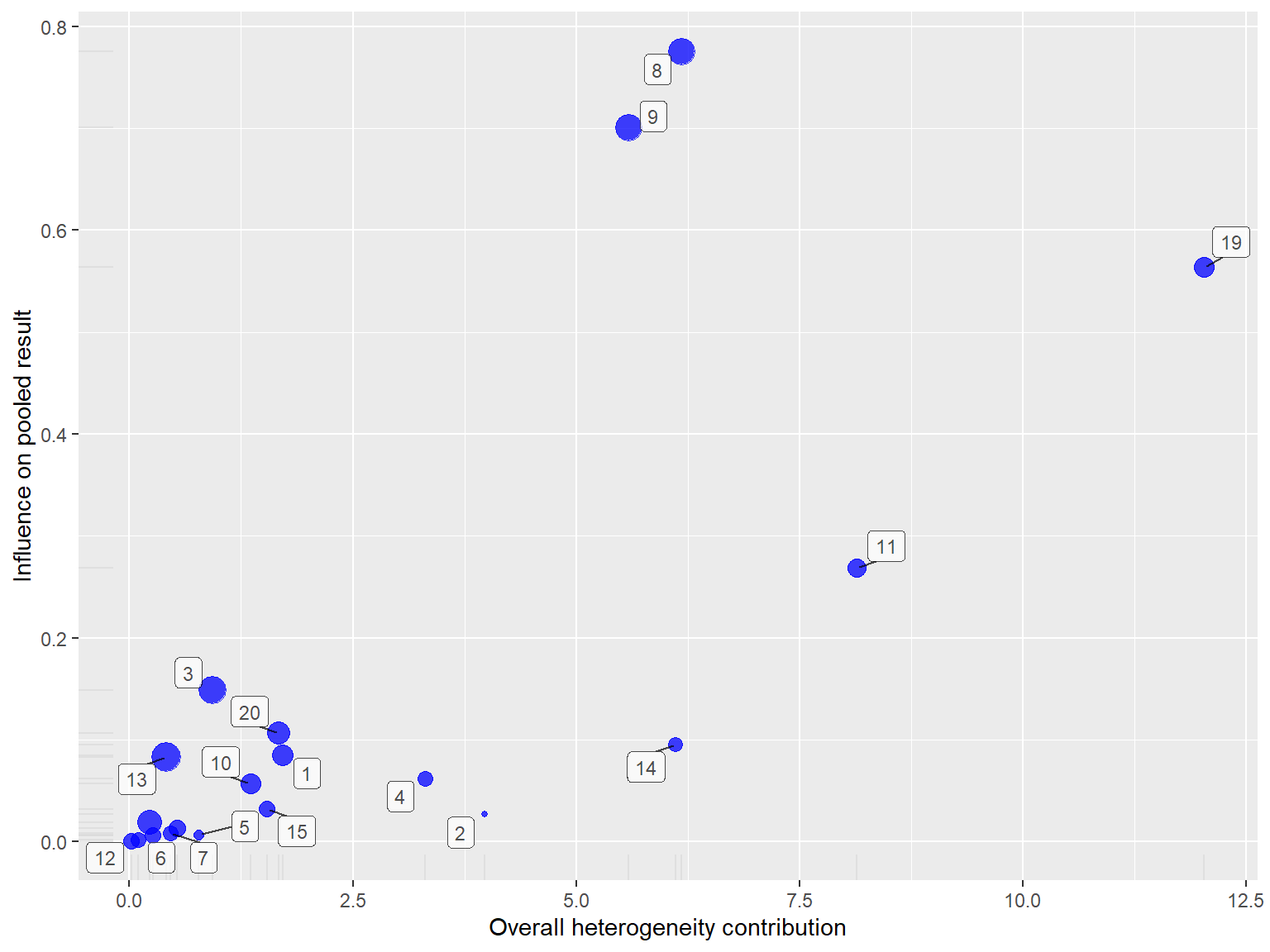


Figure S3 Outlier analysis, meta-analysis 2. ES 19 contributes disproportionately to both the variance and the pooled ES. ESs 8 and 9 mostly to the pooled ES.


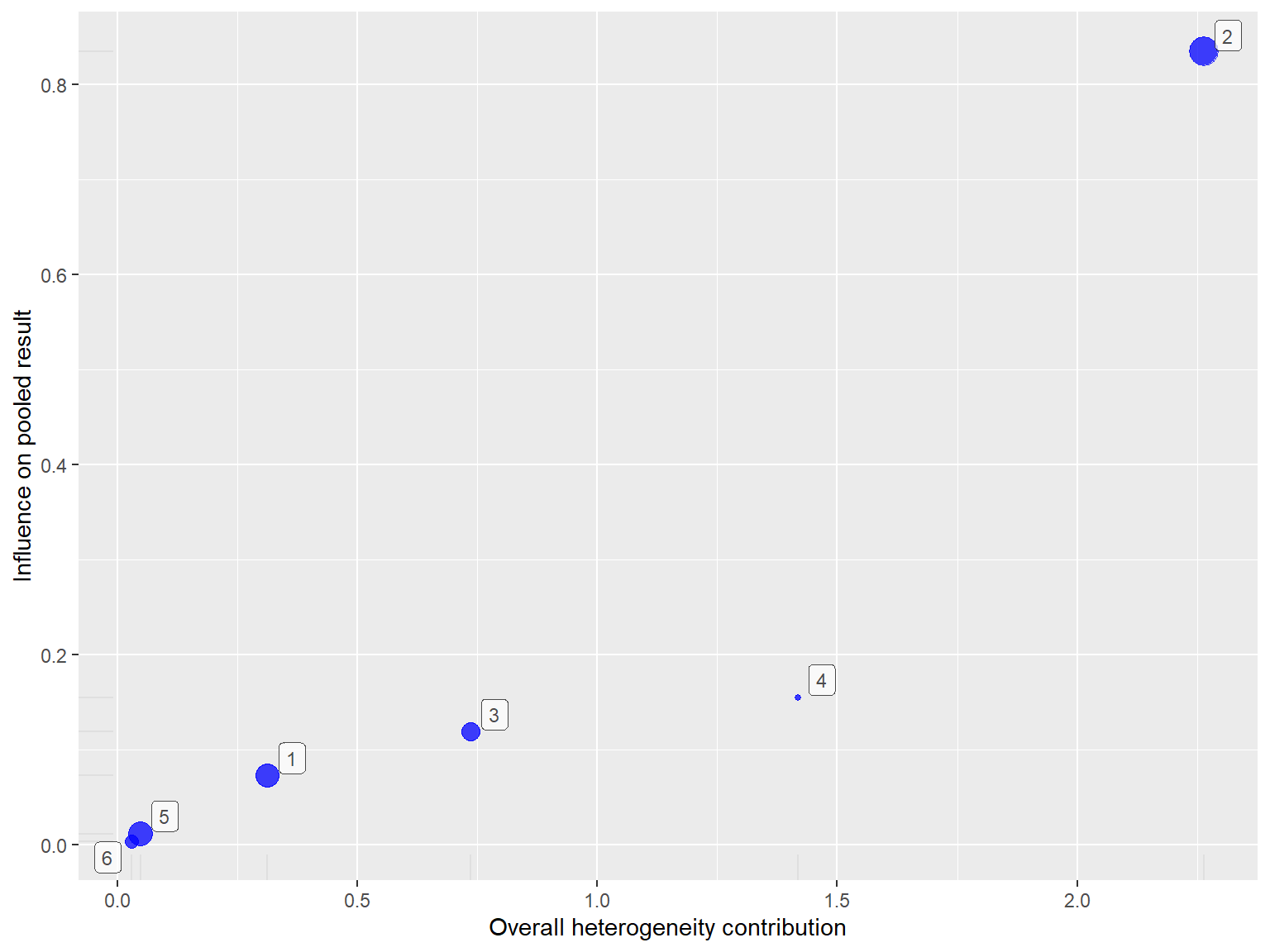


Figure S4 Outlier analysis, meta-analysis 3. ES 2 contributes disproportionately to both the variance and the pooled ES.
